# Budd‐Chiari syndrome on background of JAK2V617F mutation with no hematologic abnormalities

**DOI:** 10.1002/ccr3.4668

**Published:** 2021-09-13

**Authors:** Ammara Bint I Bilal, Fateen Ata, Mohamed Abdelrazek, Mohamed A. Yassin

**Affiliations:** ^1^ Department of Radiology Hamad Medical Corporation Doha Qatar; ^2^ Department of Internal Medicine Hamad Medical Corporation Doha Qatar; ^3^ Department of Medical Oncology/Hematology National Centre for Cancer Care and Research Hamad Medical Corporation Doha Qatar

**Keywords:** Bud‐Chiari syndrome, hepatic vein thrombosis, JAK 2 mutation, myeloproliferative disease

## Abstract

Whenever considering idiopathic Budd‐Chiari syndrome, consider the possibility of JAK2 mutation even if clinical parameters are within normal range.

## Graphical abstract

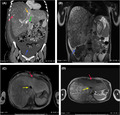

We present the clinical image of a young woman without comorbidities, who presented with massive tender hepatosplenomegaly diagnosed via CT as Budd‐Chiari syndrome (BCS). An extensive workup was done to determine the underlying cause of her presentation, and she was found to have JAK2V617F mutation on a background of normal peripheral counts and bone marrow biopsy. Worsening liver function and repeated failure at transjugular intrahepatic portosystemic shunt (TIPS) ultimately necessitated liver transplantation.

A 24‐year‐old woman presented with progressive abdominal pain and was found to have thrombosed hepatic veins, patent portal veins, and hepatosplenomegaly consistent with acute BCS with a liver span of 27 cm (Figure 1). Coagulation studies and autoimmune workup proved inconsequential in diagnosing the underlying cause (Table 1). A positive JAK2 mutation was identified even though her blood counts were within normal range and bone marrow biopsy showed hypocellularity with trilineage hematopoiesis; she was diagnosed with occult/latent myeloproliferative disorder. TIPS was attempted three times over a few months by IR but resulted in repeated occlusion while suffering multiple bouts of spontaneous bacterial peritonitis. The patient underwent liver transplantation 4.5 years after her initial presentation.

**TABLE 1:** Laboratory investigations of the patient on diagnosis, 2‐year and 4‐year follow‐ups

Parameters	Normal range	On diagnosis (2016)	After 2 years (2018)	After 4 years (2020)
Platelets	150–450 × 10^3^/L	220	203	258
WBCs	4.5–11.0 × 10^9^/L	7.3	8.1	10.0
RBCs	4.7–6.1 million cells/µl	4.1	4.3	4.3

**FIGURE 1:**
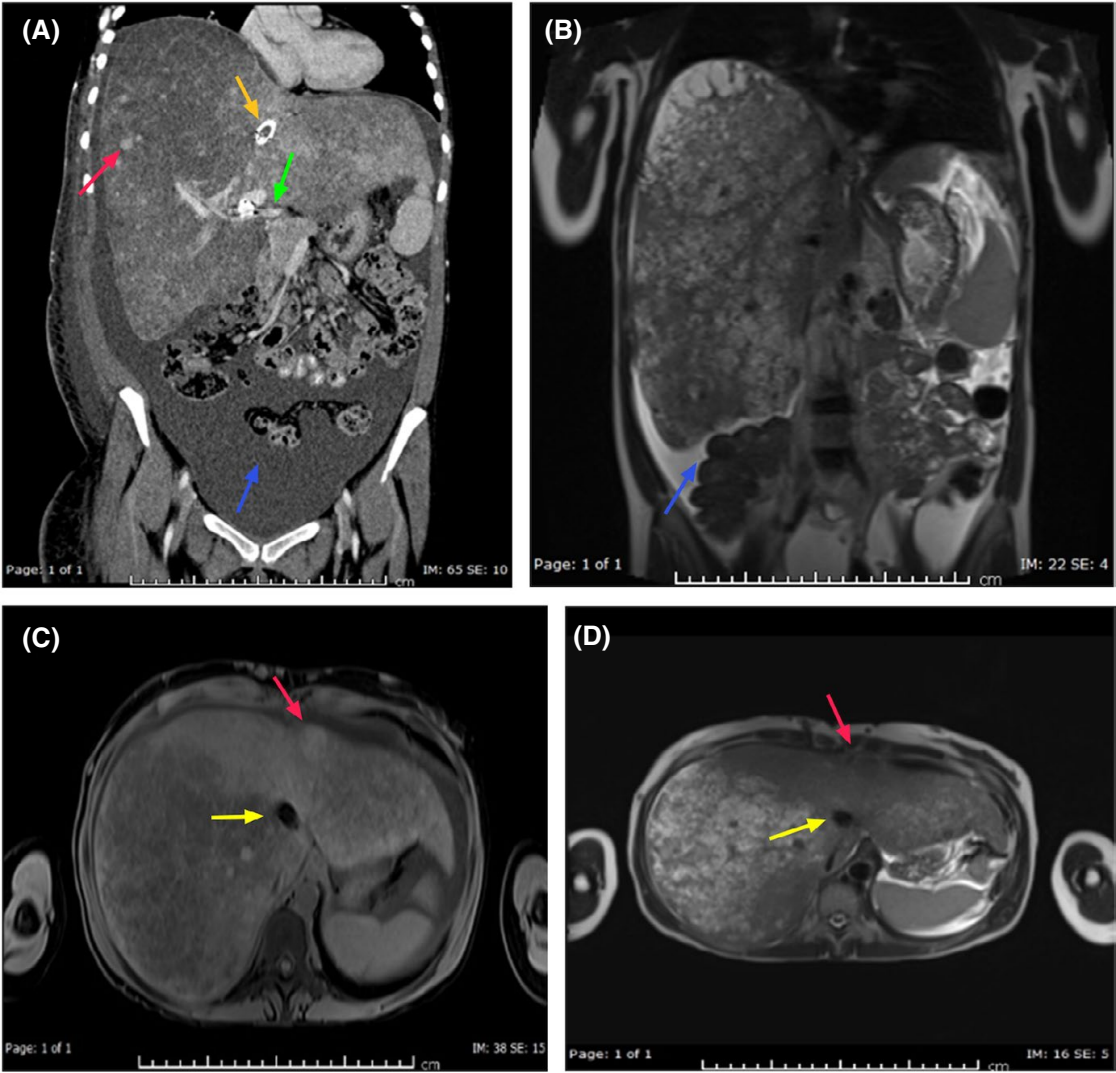
(A) Coronal CT scan of the abdomen with oral and intravenous contrast showing markedly enlarged liver with the span of the right lobe 28 cm, multiple liver nodules (red arrow), moderate ascites (blue arrow), patent portal vein (green arrow) and Transjugular intrahepatic portosystemic shunt (TIPSS) (yellow arrow). (B) Coronal MRI of the abdomen T2 WI. (C) Axial T1 WI of the abdomen. (D) Axial T2 WI of the abdomen showing enlarged heterogeneous liver with multiple nodules (red arrow), ascites (blue arrow), and TIPSS (yellow arrow)

Budd‐Chiari syndrome is the obstruction of the hepatic venous outflow tract due to the hepatic vein and/or suprahepatic inferior vena cava thrombosis.^1^ Philadelphia negative myeloproliferative neoplasms are reported as the most frequent underlying prothrombotic factors in patients with BCS (30%–50%). It is not uncommon for these patients to lack the characteristic diagnostic feature of increased peripheral blood counts, possibly due to congestion and blood accumulation in the liver and spleen. There is limited literature regarding the correlation of the prevalence and prognosis of solitary JAK2V617F in BCS patients. Among the available data, the prevalence is 6.5%, out of which 41% develop characteristic laboratory morphological features within 0.7–7 years after diagnosis.^1^

Clinicians must consider the possibility of JAK2V617F mutations (occult MPN) in patients with BCS.

## CONFLICT OF INTEREST

None declared.

## AUTHOR CONTRIBUTIONS

AB involved in methodology, literature review, manuscript writing, image selection, and revisions in the manuscript. FA performed literature review and manuscript writing. MA performed Literature review, image selection and finalization, and image legends. MY involved in supervision, manuscript writing, and revisions. All authors reviewed and approved the final version of the manuscript.

## ETHICAL APPROVAL

This work was approved by Medical Research Center (MRC) Qatar before submission.

## Data Availability

Available upon request.
